# Toothache

**Published:** 1872-10

**Authors:** J. H. Payne

**Affiliations:** Middletown, Ohio


### Toothache.

BY J. H. PAYNE, MIDDLETOWN, OHIO.

We will first speak of the cause of toothache, 2dly of its development, and 3dly of the treatment of it. We will speak of these in the order indicated.

1st. Simple toothache is caused by the uncovering of the pulp by decay of tooth substance, so that in masticating the food the pulp is no longer protected from foreign substances, but is wounded, exposed to the air, and injured by the fluids of the mouth, either of which, or all, more or less, go to superinduce disease. 2nd. Its further development. If not relieved, the inflammation extends to the periosteum, and soreness and elongation of the tooth is experienced. This may be determined by striking it gently with the end of an excavator. If the tooth springs with a dull sound, the periosteum is thickened, and the third stage, or alveolar abscess will ensue, if not prevented. If the sound is sharp and clear, and no pain results from the striking of the tooth, the inflammation is yet within the tooth, and is more easily relieved. The third stage, or alveolar abscess, is diagnosed by thickening of the alveolar process, extreme redness of the gum, swelling of that part of the face, and apparent looseness of the tooth.

First. The treatment of it. If simple toothache, the first thing we do is to clean the cavity of foreign and carious matter, being careful not to wound the exposed pulp. Then carefully enlarge the opening to the pulp chambers. This we do in two ways. First by a burr drill as large as can be introduced to the cavity, bearing upon its anterior surface a half drop of carbolic acid; with this we often succeed in cutting away the jagged edges of the cavity against which the swollen pulp has been forced and by which wounded. If the drill cannot be borne by the patient, we take any sort of good cutting instrument, a hatchet or hoe-shaped excavator, and begin back at the sides of the floor of the cavity and cut toward the pulp, relieving thereby the pressure of the dentinal wall upon the swollen pulp, often procuring great relief in this way before we come to application of remedies. If the pulp have a healthy pink appearance and succumb to the treatment, we propose to save it alive. If it have a purple appearance, strangulation has already ensued, and its extirpation is accordingly determined upon. To begin with the conservative treatment. We apply a very small bit of cotton moistened with iodine tincture and carbolic acid, half and half, previously prepared, and gently held in apposition by a bit of cotton secured by another piece either waxed or gummed with sandarach varnish. This may be left in several hours without harm, though a quarter or half hour is quite sufficient. We use a cream-like solution of oxychloride of zinc for a temporary filling. Upon the removal of the cotton, there should be no appearance of blood or escaping serum from the pulp. The surface being entirely dry, the os-artificial should be applied without pressure, in a buttery form, supported by little blocks of tin foil or bits of spunk or cotton, and allowed to harden before the remaining portion of the filling is placed. If the patient lives conveneintly near to the office, an appointment for a day or two distant may be made, but an hour or two will usually suffice to determine whether to proceed or to retrace the steps taken. In one case pain continued for two hours after the cement was applied, and we began to fear that we should have to remove the oxychloride and devitalize the pulp, extirpate and fill by the heroic, radical way, but the pulp began to be reconciled to its new quarters, finding pulse room; quiet and ease reigned, where a little while ago an enemy had caused quite a rumpus.

Second. Treatment of the second stage. We have now periostitis. If the usual remedies fail, and the patient will submit, we extract the tooth, that is, if it be one of the ten anterior teeth, and usually find an infiltrated condition of the periosteum. In a case we had sixteen months since, we had filled the tooth, leaving the pulp quite alive. About two hours afterward soreness and pain set in; lady 14 years of age; right lateral superior incisor; yielded to have it extracted. We found extravasation begun, removed the thickened viscid substance from the apex, painted root with carbolic acid undiluted; replaced the tooth in its alveoler process, and in one hour it was comfortable, and has occasioned no trouble since. We proceed in this way whether the tooth has been filled or not. We think periostitis is usually caused by irritation, chilled or wounded blood corpuscles may produce it; a blow upon the tooth, arsenious acid, or pressure, or a broken particle of broach, or presence of dead tissue, any one or a combination of these may cause peridontitis. But if the patient will not allow the extraction of the tooth for the displacement of the cause of the disease, and the replacement of the tooth, we, secondly, undertake its cure by the following plan: We apply a wedge-shaped instrument like a taper-drill, or any instrument improvised for the purpose, to the labial border of the alveolar process, penetrating between the gum and alveolar wall, and raise the *plate* of the alveolar process, so as to liberate the pent-up putrescent gas. Very many bubbles will follow the removal of the instrument, and in many cases has brought the desired relief, and enabled, by topical treatment, the powers of the system to effect a cure. If the tooth has not been plugged, we extirpate the pulp; if in a dying condition, which may be known by its color being dark (pink if alive and healthy). If dark, we apply a little arsinous paste prepared for that purpose by the Dental Depot proprietors, about the size of a mustard seed. The cavity of course has been well cleaned, in order to know the color and condition of the pulp; and let me say that no one is justified, by my judgment, in applying arsenic to a healthy tooth pulp. The directions in the former part of this paper for the capping of a healthy pulp will give patient and dentist far less trouble and pain than the application of arsenious paste.

Two hours is usually sufficient time to devitalize—we do not want to kill either the pulp or periosteum, or nerve fibrils. We simply need to obtund the sensibility of the pulp, so that we can remove it without too great pain to the patient. We use a barbed broach for that purpose, and we must be very careful of two things, 1st, not to push through the foramen any lymph, nor, 2d, to break off our broach in the apex. Should we do one or the other, or both, two or three things are likely to ensue: 1st, peridontitis, with rapid tumefaction and alveolar abscess; if not relieved, the unwillingness of our patient longer to submit to our endeavors to relieve him instrumentally, or we must come down and yield and extract the tooth, and we bear the chagrin and the patient the loss of the tooth. But this need not occur; we should not press the broach so deeply as to thrust through the foramen. After carefully clearing the pulp canal, time may be given, a day or two, for nature to restore quiet to the parts. When no longer sore, the tooth may be filled. The pulp canal may be filled either with gold, tin or oxychloride. We have no preference, except where gold is used for the crown filling, then we prefer gold for the canal too.

But, lastly, alveolar abscess. How shall we treat it. This has been anticipated in part. First, as in acute periostitis an opening must be made.

1st The corpse of the deceased dental pulp must be removed. The tooth must be disinfected. If an opening has been made through the anterior alveolar process, use that as a lead to find the sac, at the foramen, and direct away the cushion, now no longer to be needed as an absorbent to arrest the putresent gas, which has begun to be generated by the pulp.

The principal of carbolic acid should be used in disinfecting the parts, but active surgery is not to be neglected in cutting away the diseased membranes. If the patient will submit to extraction, that will afford the dental surgeon an easier and better opportunity to remove the subgorged vessel, apply the disinfectant, and render more certain the cure, always replacing the tooth, which may be done, even with a molar whose roots diverge one-quarter of their radius, by cutting off the ends of the roots. Whether this regime will pay, is another thing. If the surgeon dentist have the highest good of his patent at heart, it will pay him in the inner-man to do so great a good for his fellow, and if his patient be equally appreciative of his dentist's good, he will certainly not neglect to remunerate him according to his best ability. The filling of such a tooth, after its health is restored, is the same as in the former case.

